# Association of cannabis and/or opioid with quality of life and healthcare utilization in patients with chronic pain

**DOI:** 10.3389/fpain.2022.1015605

**Published:** 2022-11-24

**Authors:** Vafi Salmasi, Lorene M. Nelson, Juliette Hong, Sean C. Mackey

**Affiliations:** ^1^ Department of Anesthesiology, Perioperative and Pain Medicine, Stanford University School of Medicine, Palo Alto, CA, United States; ^2^ Department of Epidemiology and Population Health, Stanford University School of Medicine, Palo Alto, CA, United States

**Keywords:** opioid, cannabis, chronic pain, healthcare utilization, quality of life

## Abstract

**Background:**

Opioids have been commonly used to treat chronic pain, but they are associated with significant morbidity and mortality. Cannabis has been advocated as an alternative; however, a growing number of patients are now using a combination of opioid and cannabis and the impact of this combination is not well-studied.

**Aim:**

We characterized use of opioid and/or cannabis in patients with chronic pain; and compared utilization of healthcare resources.

**Methods:**

We conducted a cross-sectional study to determine if measures of physical, psychological and social functioning differed among patients according to whether they used opioids and/or cannabis. We used our learning healthcare system – CHOIR – to capture NIH Patient Reported Outcomes Measure Information System surveys, and legacy pain and treatment specific questions.

**Results:**

Patients who report use of opioid and/or cannabis experience higher levels of physical, psychological and social distress. After adjusting for inversed weight of propensity scores, they have higher odds of visiting an emergency room, staying overnight at the hospital, and visiting a physician.

**Conclusion:**

Our results show that use of opioid and/or cannabis is associated with worse baseline characteristics and outcomes. Our study however cannot determine if worse outcomes are due to the opioids and/or cannabis or simply that these patients are worse off before using opioids and/or cannabis. Thus, it is important to characterize the trajectory of these patients in a prospective longitudinal study.

## Introduction

Chronic pain is a costly disease affecting more than 100 million Americans with approximately 20 million estimated to suffer from high-impact chronic pain – or pain with *“*substantial restriction of participation in work, social, and self-care activities” (1). Chronic pain bears an estimated annual cost of $635 billion and is recognized as a disease of its own that significantly reduces quality of life (2). Unfortunately, there is a significant gap in both treatment modalities available for treatment of chronic pain, and appropriate evidence to support application of the current treatment modalities (medications, interventions, behavioral therapy, physical therapy, alternative treatment modalities, self care techniques, etc.) (2, 3). A report by Institute of Medicine (now National Academy of Medicine) called for better characterization of patients with chronic pain to develop more effective treatment strategies (2).

Long-term use of opioids, cannabis or a combination of both is common in patients with chronic pain. Prescription opioids can cause tolerance, dependence, addiction and even mortality secondary to intentional or accidental overdose, which has resulted in national crisis. Based on 2015 Vital Signs report by Centers for Disease Control and Prevention, the number of deaths caused by opioid overdose has almost quadrupled in less than two decades (4, 5). It is unclear how legalization of cannabis has affected this crisis. Synergistic effects of opioids and cannabis results in psychomotor slowing, diminished sensorium and delirium; these effects can result in morbidity and mortality because of motor vehicle accidents, falls, trauma and overdose (6–9). Initial ecologic studies in states with legalized cannabis has shown decrease in death related to opioid overdose (10–15), even though these results were not reproduced in later studies (6, 16). Moreover, synergistic effects of these substances can achieve superior analgesia in certain patients; addition of small amounts of cannabis to prescription opioids might improve outcome (17–23).

By normal scientific standards, all the evidence on how cannabis affects pain and opioid use is weak and cannot support routine use of cannabis in treating chronic pain or its effect in decreasing dose of opioids (24–32). But it has been hyped in the media to a remarkable extent; thus, many people with chronic pain now believe that cannabis will lessen their pain, will reduce their reliance on opioids, or both. Clinicians are commonly faced with questions from people with pain about cannabis, including whether to use cannabis in conjunction with prescribed opioids, whether cannabis will reduce pain, and whether cannabis will reduce reliance on opioids.

To better understand and address this problem we performed an observational, cross-sectional study to: (1) characterize baseline characteristics of patients who report using opioids and/or cannabis in our clinic; and (2) assess how use of cannabis and/or opioid affects utilization of healthcare resources.

## Methods

After approval by institutional review board (Stanford University IRB-28435), we conducted a cross sectional study (using chart review and surveys from a learning healthcare system) to characterize patients who report opioid and/or cannabis use. Based on institutional review board (IRB) recommendations, our study was exempt from obtaining informed consent. Stanford IRB evaluates each human subject study following a checklist to determine exemption from obtaining informed consent. Our research was exempt because it met the criteria for: “Chart review studies that only involve the use of data, documents, records”.

### Data collection platform

Stanford Pain Management Center has developed and implemented a registry-based, open-source learning healthcare system (LHS), Collaborative Health Outcomes Information Registry (CHOIR; http://choir.stanford.edu) since 2012. This LHS includes National Institute of Health Patient Reported Outcomes Measurement Information System (PROMIS) (33) item banks, a body map, questions about pain intensity, and pain catastrophizing scale in all surveys (initial and follow up). CHOIR uses a locally implemented computer adaptive testing engine to reduce patient survey burden, while maintaining or improving estimates of the domain of interest. In addition, initial survey includes questions about treatment history, pain experience, healthcare utilization, opioid and cannabis use. We asked patients to complete surveys before each clinical visit (initial survey the first time they complete a survey and a follow up survey for every visit after that). Multiple studies have resulted from CHOIR data, (34–42) however none have previously addressed the hypotheses outlined in this study.

### Patients

We included the initial visit for all patients with chronic pain referring to Stanford Pain Management Center who had completed the CHOIR initial survey with cannabis survey. We retrieved 15,182 initial surveys between 2012 and 2018 of which 8,869 (59.4%) patients had completed a cannabis survey.

**Figure 1:**
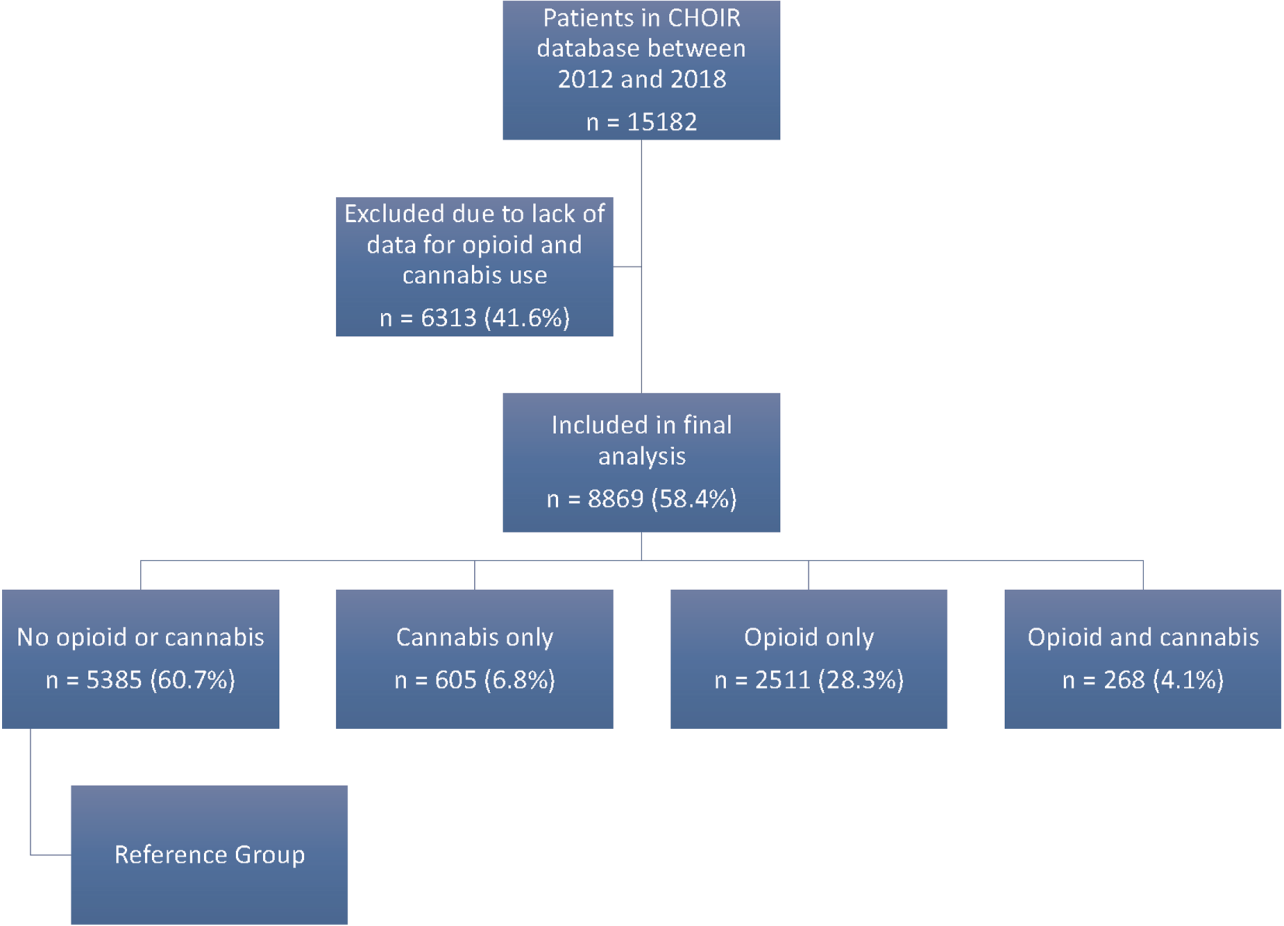
Patient flow diagram.

We then divided the patients into four groups based on their responses to questions about opiod and cannabis: (1) patient who deny use of opioid and cannabis (reference group); (2) patients who report use of cannabis only; (3) patients who report use of opioid only; and (4) patients who report use of cannabis and opioid (Figure 1).

### Measures

*Demographic variables* include patients' gender, race/ethnicity, employment status, marital status, disability status, and healthcare utilization (number of office visits, emergency room visits, and number of nights spent in a hospital within six months prior to patients' visits).

#### The patient-reported outcomes measurement information system (PROMIS)

The PROMIS measures are well-validated and widely used to assess physical and psychosocial health status in patients with chronic illnesses, including chronic pain (43–46). Detailed information about the measure development and validation is available at http://www.healthmeasures.net. The PROMIS measures are scored on a T-score metric with a mean of 50 and a standard deviation of 10. The mean of 50 is calibrated to either the mean of a United States reference sample that matched the 2,000 General Census sample with respect to age, sex, race/ethnicity, and education or to a clinically relevant population (47, 48). PROMIS CAT instruments for pain interference, physical function, fatigue, sleep disturbance, depression, anxiety, social isolation, and satisfaction with roles and activities were administered. Higher scores on each PROMIS measure generally indicate greater severity of each symptom domain. However, higher scores on PROMIS physical function indicate better physical functioning, and higher scores on satisfaction with roles and activities indicate higher satisfaction with social roles.

#### Pain intensity

Pain intensity was assessed on a numerical rating scale (NRS) using a modified PROMIS Pain Intensity scale. Respondents were asked to rate their average pain intensity over the previous 7 days on a scale of 0–10.

#### Pain catastrophizing

The Pain Catastrophizing Scale (PCS) is a 13-item questionnaire that assesses distress regarding the cognition and emotion associated with actual or anticipated pain (49). The PCS comprises three different subscales: rumination, magnification, and helplessness. Patients rate the frequency of pain-related thoughts and feelings they have in response to actual or anticipated pain using a 5-point scale that ranges from 0 = not at all through 4 = all the time. may rate each item on a 5-point Likert Scale (0 = Not at all to 4 = All the time). The PCS has a minimum score of 0 and a maximum score of 52 with higher scores indicative of more catastrophic thinking.

#### Opioid and cannabis use

Opioid and cannabis use was self-reported by patients in their CHOIR surveys. CHOIR surveys directly ask if the patients are currently using opioids and cannabis. The patients who responded to both these questions were included in the study. CHOIR initial surveys always included questions about opioid use while cannabis use questions were added later in 2015.

### Statistical analysis

We tested the hypotheses that:

*Hypothesis 1.* Patients with chronic pain who report use of opioids and/or cannabis (as compared to those not using opioids or cannabis) have worse scores for pain intensity, pain interference, pain behavior, physical function, sleep disturbance, depression, anxiety, fatigue, and social isolation.

The estimations were by multinomial logistic regression with generalized logit link for categorical variables and by generalized linear model for continuous variables with Bonferroni correction.

*Hypothesis 2.* Patients with chronic pain who report use of opioids and/or cannabis have higher healthcare utilization, as compared to those not using opioids or cannabis.

The group differences were estimated by using negative binomial models with log link on emergency room visit, hospital stay and physician visit. Because the study used observational data, propensity weighting was used to control for differences on measured covariates between the groups. Models were weighted by the stabilized inverse probability of receiving the treatment that the patient actually received. Covariates used for propensity adjustment included age, gender, race, ethnicity, body map widespread pain, PROMIS measures, physical and psychological comorbidities. To minimize the influence of extreme outlier weights, the stabilized weights were truncated to 10 if the stabilized weight is greater than 10 (50–52). All analyses were conducted using SAS Enterprise Guide Version 7.1 (SAS Institute Inc., Cary, NC, USA.).

## Results

We retrieved 15,182 initial surveys between 2012 and 2018 of which 8,869 (58.4%) patients had answered to using or not using opioid and/or cannabis. We compared the patients who have and have not completed cannabis questions. The baseline characteristics (age, sex, race) and healthcare utilization indices (emergency room visit, overnight hospital stay, number of physician visits) were similar between these groups (Appendix, Table A1). However, patients who have not completed cannabis questions had higher pain scores and worse PROMIS measures (Appendix, Table A2). Table 1 shows the distribution of pain diagnoses and comorbidities between these patients.

**Table 1:** Demographic and clinical characteristics of patients.

Variable	Total Sample (*n* = 8869)	No Cannabis + No Opioid (*n* = 5385)	Cannabis Only (*n* = 605)	Opioid Only (*n* = 2511)	Opioid + Cannabis (*n* = 368)	*P*-value
Age, Mean (SD)	51.8 (16.4)	51.2 (16.7)	47.1 (16.9)	54.5 (15.5)	49.5 (15.3)	<.001**
Gender						
Female	7,669 (67.1%)	4,916 (67.8%)	416 (61.9%)	2,096 (67.7%)	241 (58.2%)	<.001**
Male	3,758 (32.9%)	2,330 (32.2%)	256 (38.1%)	999 (32.3%)	173 (41.8%)	<.001**
Race						
Asian	1,067 (9.3%)	857 (11.8%)	20 (3.0%)	179 (5.8%)	11 (2.7%)	<.001**
African American	420 (3.7%)	239 (3.3%)	28 (4.2%)	135 (4.4%)	18 (4.3%)	0.05*
White	7,417 (64.9%)	4,528 (62.5%)	480 (71.4%)	2,119 (68.5%)	290 (70.0%)	<.001**
Native American/Pacific Islander	140 (1.2%)	79 (1.1%)	7 (1.0%)	46 (1.5%)	8 (1.9%)	0.20
Others	2,383 (20.9%)	1,543 (21.3%)	137 (20.4%)	616 (19.9%)	87 (21.0%)	0.45
Ethnicity						
Hispanic/Latino	1,375 (12.0%)	847 (11.7%)	80 (11.9%)	391 (12.6%)	57 (13.8%)	0.38
Non-hispanic	1E4 (88.0%)	6,399 (88.3%)	592 (88.1%)	2,704 (87.4%)	357 (86.2%)	0.38
Pain Region						
Headache/Facial Pain	4,775 (41.8%)	3,325 (45.9%)	281 (41.8%)	1,031 (33.3%)	138 (33.3%)	<.001**
Neuropathic Pain	5,739 (50.2%)	3,587 (49.5%)	267 (39.7%)	1,687 (54.5%)	198 (47.8%)	<.001**
Musculoskeletal Pain	7,925 (69.4%)	4,890 (67.5%)	407 (60.6%)	2,354 (76.1%)	274 (66.2%)	<.001**
Multiple/Other Diagnosis	5,530 (48.4%)	3,154 (43.5%)	309 (46.0%)	1,820 (58.8%)	247 (59.7%)	<.001**
Fibromyalgia	3,056 (26.7%)	1,937 (26.7%)	168 (25.0%)	853 (27.6%)	98 (23.7%)	0.25
Visceral Pain	3,294 (28.8%)	2,088 (28.8%)	175 (26.0%)	913 (29.5%)	118 (28.5%)	0.36
Cancer Pain	188 (1.6%)	60 (0.8%)	14 (2.1%)	95 (3.1%)	19 (4.6%)	<.001**
Complex Regional Pain Syndrome	1,090 (9.5%)	661 (9.1%)	49 (7.3%)	338 (10.9%)	42 (10.1%)	0.006*
Comorbidity						
Congestive Heart Failure	433 (3.8%)	253 (3.5%)	14 (2.1%)	153 (4.9%)	13 (3.1%)	<.001**
Diabetes-uncomplicated	1,084 (9.5%)	618 (8.5%)	53 (7.9%)	370 (12.0%)	43 (10.4%)	<.001**
Diabetes-complicated	551 (4.8%)	298 (4.1%)	32 (4.8%)	193 (6.2%)	28 (6.8%)	<.001**
Liver Disease	821 (7.2%)	489 (6.7%)	30 (4.5%)	260 (8.4%)	42 (10.1%)	<.001**
Lymphoma	162 (1.4%)	91 (1.3%)	10 (1.5%)	53 (1.7%)	8 (1.9%)	0.26
Metastatic Cancer	367 (3.2%)	176 (2.4%)	20 (3.0%)	141 (4.6%)	30 (7.2%)	<.001**
Obesity	1,664 (14.6%)	966 (13.3%)	83 (12.4%)	554 (17.9%)	61 (14.7%)	<.001**
Chronic Pulmonary Disease	1,995 (17.5%)	1,222 (16.9%)	96 (14.3%)	584 (18.9%)	93 (22.5%)	<.001**
Peripheral Vascular Disorder	716 (6.3%)	431 (5.9%)	31 (4.6%)	231 (7.5%)	23 (5.6%)	0.007*
Renal Failure	561 (4.9%)	299 (4.1%)	24 (3.6%)	213 (6.9%)	25 (6.0%)	<.001**
Solid Tumor Without Metastasis	1,038 (9.1%)	609 (8.4%)	48 (7.1%)	330 (10.7%)	51 (12.3%)	<.001**
Valvular Disease	565 (4.9%)	350 (4.8%)	27 (4.0%)	172 (5.6%)	16 (3.9%)	0.18
Mental Health						
Anxiety Disorder	3,046 (26.7%)	1,867 (25.8%)	177 (26.3%)	887 (28.7%)	115 (27.8%)	0.02*
Bipolar Or Manic Disorder	460 (4.0%)	268 (3.7%)	39 (5.8%)	134 (4.3%)	19 (4.6%)	0.04*
Concussion (Mild Tbi)	143 (1.3%)	110 (1.5%)	4 (0.6%)	25 (0.8%)	4 (1.0%)	0.01*
Depression	3,005 (26.3%)	1,766 (24.4%)	179 (26.6%)	926 (29.9%)	134 (32.4%)	<.001**
Post Traumatic Stress Disorder	350 (3.1%)	179 (2.5%)	29 (4.3%)	121 (3.9%)	21 (5.1%)	<.001**
Schiphrenia And Other Psychotic Disorder	188 (1.6%)	111 (1.5%)	9 (1.3%)	59 (1.9%)	9 (2.2%)	0.40
Intracranial Injury Excluding Concussion	194 (1.7%)	136 (1.9%)	12 (1.8%)	38 (1.2%)	8 (1.9%)	0.13
Sleep Disorder Excluding Narcolepsy	3,239 (28.3%)	2,047 (28.3%)	181 (26.9%)	882 (28.5%)	129 (31.2%)	0.51
Alcohol Related Disorder	338 (3.0%)	184 (2.5%)	21 (3.1%)	110 (3.6%)	23 (5.6%)	0.001*
Substance Abuse Other Than Opioid	786 (6.9%)	352 (4.9%)	75 (11.2%)	291 (9.4%)	68 (16.4%)	<.001**
Suicidal Ideation	157 (1.4%)	96 (1.3%)	11 (1.6%)	47 (1.5%)	3 (0.7%)	0.54
Suicide Attempt And Self Inflicted Injury	47 (0.4%)	23 (0.3%)	5 (0.7%)	19 (0.6%)	0 (0.0%)	0.12
Opioid Use Disorder	725 (6.3%)	263 (3.6%)	27 (4.0%)	382 (12.3%)	53 (12.8%)	<.001**
Pain Measure						
No. Wide-spread Pain, Mean (SD)	12.2 (12.7)	10.9 (11.6)	14.7 (15.0)	14.1 (13.6)	15.3 (14.9)	<.001**
Pain Catastrophizing Scale, Mean (SD)	22.0 (12.8)	20.9 (13.0)	23.2 (12.4)	23.5 (12.4)	26.0 (12.1)	<.001**
Pain Intensity - Average, Mean (SD)	5.5 (2.3)	5.1 (2.3)	5.3 (2.2)	6.1 (1.9)	6.2 (1.8)	<.001**
PROMIS Measure						
Anger	49.3 (10.4)	48.4 (10.4)	51.3 (10.3)	50.2 (10.2)	53.1 (9.9)	<.001**
Pain Interference	63.6 (7.8)	62.4 (8.2)	64.2 (7.3)	65.9 (6.2)	67.0 (5.8)	<.001**
Pain Behavior	58.3 (5.4)	57.5 (6.0)	58.9 (4.7)	59.7 (3.6)	60.6 (3.3)	<.001**
Fatigue	58.2 (10.4)	56.9 (10.6)	59.1 (10.5)	60.4 (9.4)	62.1 (9.0)	<.001**
Sleep Disturbance	56.0 (9.4)	54.8 (9.5)	56.9 (9.3)	58.0 (8.8)	59.4 (8.6)	<.001**
Depression	53.7 (10.0)	52.6 (10.1)	55.2 (9.6)	55.2 (9.8)	57.7 (8.7)	<.001**
Anxiety	54.7 (9.9)	54.0 (10.0)	56.1 (9.7)	55.8 (9.6)	58.1 (8.9)	<.001**
Emotional Support	51.1 (9.5)	51.1 (9.5)	50.9 (9.3)	51.1 (9.5)	51.4 (8.9)	0.88
Satisfaction Role	42.9 (9.9)	44.5 (10.0)	41.7 (9.8)	40.3 (9.2)	38.5 (8.0)	<.001**
Sleep Impairment	55.7 (10.1)	54.8 (10.3)	56.9 (9.9)	57.3 (9.4)	59.0 (9.2)	<.001**
Social Isolation	47.2 (9.5)	46.4 (9.5)	49.6 (9.5)	48.0 (9.5)	50.7 (9.2)	<.001**

*p-value < 0.05.

**p-value < 0.001.

PROMIS, Patient-Reported Outcomes Measurement Information System; SD, Standard Deviation; CI, Confidence Interval; PCS, Pain Catastrophizing Scale.

The *p* values were calculated by fitting in generalized logit model for categorical variables and generalized linear model for continuous variables with bonferroni correction.

Table 1 also summarizes the baseline characteristics of the patients in final analysis (8,869 patients). Opioid and/or cannabis use is associated with worse scores for pain intensity, pain catastrophizing, pain interference, pain behavior, fatigue, depression, anxiety, anger, sleep quality, social isolation, and satisfaction with social roles and activities. Emotional support was the only measure that was not significantly different between the groups.

We observed a similar pattern in models weighted by inversed propensity scores (Table 2). Comparing with patients who denied use of opioid and cannabis, opioid and/or cannabis users reported more widespread pain; higher pain intensity; and worse anger, anxiety, depression, fatigue, pain behavior, pain interference, satisfaction with social roles, social isolation, sleep impairment, and sleep disturbance. Similarly, the differences in emotional support were not significant in either group after weighting for inverse propensity scores.

**Table 2:** Differences in pain intensity, body Map areas and PROMIS measures by cohort^a^.

Outcome	Cannabis Only^c^		Opioid Only^c^		Opioid + Cannabis^c^	
	Estimate (CI)	*P*-value	Estimate (CI)	*P*-value	Estimate (CI)	*P*-value
No. Wide-spread Pain	2.90 (1.86, 3.94)	<.001^c^	3.01 (2.42, 3.59)	<.001^c^	4.80 (3.50, 6.09)	<.001^c^
Pain Intensity - Average	0.11 (−0.07, 0.28)	0.23	0.80 (0.71, 0.89)	<.001^c^	0.75 (0.53, 0.97)	<.001^c^
Pain Catastrophizing Scale	0.80 (−0.22, 1.81)	0.12	2.30 (1.76, 2.85)	<.001^c^	3.47 (2.21, 4.74)	<.001^c^
PROMIS Anger	2.29 (1.46, 3.11)	<.001^c^	1.55 (1.12, 1.99)	<.001^c^	3.35 (2.32, 4.38)	<.001^c^
PROMIS Anxiety	1.37 (0.59, 2.16)	0.001^b^	1.41 (0.99, 1.82)	<.001^c^	3.25 (2.27, 4.23)	<.001^c^
PROMIS Depression	1.74 (0.95, 2.53)	<.001^c^	2.03 (1.61, 2.45)	<.001^c^	4.20 (3.21, 5.19)	<.001^c^
PROMIS Emotional Support	−0.21 (−0.97, 0.54)	0.58	0.21 (−0.19, 0.62)	0.30	0.84 (−0.10, 1.79)	0.08
PROMIS Fatigue	1.40 (0.58, 2.21)	0.001^b^	2.98 (2.55, 3.42)	<.001^c^	5.15 (4.13, 6.17)	<.001^c^
PROMIS Pain Behavior	1.05 (0.64, 1.46)	<.001^c^	1.90 (1.69, 2.12)	<.001^c^	2.65 (2.14, 3.17)	<.001^c^
PROMIS Pain Interference	1.08 (0.48, 1.68)	<.001^c^	3.05 (2.73, 3.36)	<.001^c^	4.18 (3.42, 4.93)	<.001^c^
PROMIS Satisfaction Role	−2.39 (−3.17, −1.62)	<.001^c^	−3.52 (−3.93, −3.10)	<.001^c^	−5.95 (−6.91, −4.98)	<.001^c^
PROMIS Sleep Disturbance	1.31 (0.57, 2.05)	0.001^b^	2.92 (2.53, 3.31)	<.001^c^	3.87 (2.94, 4.80)	<.001^c^
PROMIS Sleep Impairment	1.22 (0.42, 2.02)	0.003^b^	2.43 (2.00, 2.85)	<.001^c^	3.33 (2.34, 4.33)	<.001^c^
PROMIS Social Isolation	2.58 (1.83, 3.34)	<.001^c^	1.40 (1.00, 1.81)	<.001^c^	3.92 (2.97, 4.86)	<.001^c^

CI, Confidence Interval; PROMIS, Patient-Reported Outcomes Measurement Information System.

^a^ The models were weighted by stabilized inverse probability of receiving the group assignment that the patient received.

^b^ *P*-value < 0.05.

^b^ *P*-value < 0.001.

^c^ Reference group: neither opioid nor cannabis.

The patients with chronic pain who reported use of opioid and/or cannabis also reported higher use of healthcare resources as summarized in Table 3. After weighting for inverse propensity score, use of opioids and cannabis significantly increased the odds of visiting a physician office (45% more likely with confidence interval of 0.36–0.54), visiting an emergency room (59% more likely with confidence interval of 0.41–0.78), and stating at the hospital overnight (92% more likely with confidence interval of 0.63–1.22). We observed the same trend for patient who reported use of opioid only or cannabis only, but it did not meet the threshold of statistical significance at *α *= 0.05 for overnight hospital stay in patients who reported use of cannabis only.

**Table 3:** Differences in health utilization by cohort^a^.

Outcome	Cannabis Only^d^		Opioid Only^d^		Opioid + Cannabis^d^	
	Estimate (CI)	*P*-value	Estimate (CI)	*P*-value	Estimate (CI)	*P*-value
Physician Visit	0.30 (0.22, 0.37)	<.001^c^	0.31 (0.27, 0.36)	<.001^c^	0.45 (0.36, 0.54)	<.001^c^
Emergency Room Visit	0.34 (0.20, 0.50)	<.001^c^	0.48 (0.40, 0.57)	<.001^c^	0.59 (0.41, 0.78)	<.001^c^
Hospital Stay	0.27 (0.02, 0.52)	0.03^b^	0.74 (0.60, 0.88)	<.001^c^	0.92 (0.63, 1.22)	<.001^c^

CI, Confidence Interval.

^a^ The models were weighted by stabilized inverse probability of receiving the group assignment that the patient received.

^b^ *P*-value < 0.05.

^c^ *P*-value < 0.001.

^d^ Reference group: neither opioid nor cannabis.

## Discussion

Our results highlight that patients who report use of opioid and/or cannabis experience higher level of pain intensity and pain catastrophizing; their pain interferes more with their life and causes more pain related behavior. These patients are more fatigued, depressed, anxious and angry; they have poorer sleep and are not as satisfied with their social roles and activities. Moreover, they utilize higher level of healthcare resources.

Significant implications of opioid crisis and deaths caused by opioid related overdose have encouraged pain specialists to look for better alternatives. Some have advocated use of cannabis as a substitute relying on population-based studies showing decrease in fatalities related to opioids in states where cannabis was legalized (10–15); however, more widespread use of cannabis has expanded the population of patients who use a combination of these two medications. In our study, 476 patients reported use of both opioid and cannabis despite recommendation from nearly all prescribers not to use these medications simultaneously (which is reflected in most patient-clinician agreements for long-term treatment with controlled medications). Prior studies have shown the synergistic effect of opioid and cannabis both in analgesia and side effects (6–9, 17, 18). But data about characterization and outcome of these patients is lacking; our study addresses this gap in knowledge.

A few studies have evaluated patients who use a combination of opioids and cannabis. These studies reported higher incidence of depression, anxiety, tobacco use, and history of opioid abuse/misuse in patients with chronic pain (53–55). These studies did not assess other characteristics of the patients, did not include a group of patients that did not use opioid or cannabis, and had smaller number of patients (317, 880 and 888 patients) (53–55). Our observations verify these results but in a larger population.

Vigil et al*.* reported that use of cannabis in patients with chronic opioid use can result in successful weaning of opioids. However, their sample size was relatively small (37 cases and 29 controls) (56). These results slightly differ from findings of Shah et al*.* who followed patients prospectively in a pain rehabilitation program. Similar to our results, they reported worse baseline outcomes for patients with positive urine toxicology screen for cannabis; however, the trajectory of the groups were similar in their pain rehabilitation program (57).

We also found out that use of a combination of opioid and cannabis is associated with higher incidence of visiting emergency room, staying overnight at the hospital and also more physician visits (after adjusting for age, sex, pain intensity and PROMIS measures). Patients with chronic pain who reported using opioid or a combination of opioid and cannabis similarly reported higher incidence of visiting emergency room or staying overnight in the hospital. A similar trend (but to a smaller magnitude and not statistically significant) was also observed in patients who reported use of cannabis. These findings suggest that use of opioid is a more important risk factor than cannabis for visiting emergency room or requiring an overnight stay in the hospital. For physician visits, patients who used opioids or cannabis had a similarly increased number of physician visits, while those who used a combination of opioid and cannabis had a larger number of physician visits, suggesting that the combination plays an important role than either substance by itself. It is important to emphasize that the observed odds ratios are statistically significant but only modest.

Our data represents a large number of patients (8,869 patients) even though less than 10% of them reported using a combination of opioid and cannabis. This large number of study subjects gave us the ability to study multiple independent variables in multiple groups. It also provided appropriate power to adjust for all these variables when assessing the health care utilization consequences of using a combination of opioid and cannabis. However, the nature of our data does not allow to make a distinction between effects of opioid or cannabis in the patients who reported use of both.

The other strength of our study is use of comprehensive array of patient-reported outcomes across multiple domains of physical, psychological and social functioning. Pain is an “experience” based on the definition by International Association for Study of Pain (IASP) (58). To better understand this experience and its implications, it is important to record and study the outcome that patients with chronic pain report (3, 59, 60). Accordingly, our study focuses on validated measures of patient reported outcomes across all these aspects of functioning.

Clearly, our study has certain limitations. *First*, we performed a retrospective cross-sectional study. We cannot make any causal inferences or even assess the temporality of associations found. This study is an initial exploratory study, and our data cannot show if differences between the groups are secondary to use of opioids and/or cannabinoids. *Second,* opioid and cannabis were recorded based on patients’ self-report. Considering the social sensitivity of reporting about these two substances, we expect some degree of under-reporting by the patients. *Third*, there were two sources of missing data: (1) patients who do not complete CHOIR surveys; and (2) patient who skip the sections about cannabis use or were not offered this questionnaire (42.7% of the patients who have completed CHOIR initial survey). We expect the patients who completed CHOIR surveys to be different from the patient who did not; however, we do not expect this issue to affect the internal validity of our findings but could potentially affect the generalizability of our findings. The patients who did not complete the cannabis survey (i.e., who chose not to complete or were not offered the survey) were similar to the patients in our analysis in baseline demographics (age, sex and race) and healthcare utilization indices (data not shown), but they did have higher pain intensity and worse PROMIS measures at baseline. Given these differences, we can consider two extreme scenarios and the effects they would have on our conclusions: *(1) Majority of patients with missing cannabis questionnaire do not use opioid and/or cannabis.* If this were this case, we expect that having excluded these patients from our sample would have decreased the magnitude of difference in all comparisons done in aim one or make them statistically non-significant. *(2) Majority of patients with missing cannabis questionnaire use opioid and/or cannabis.* If this were the case, we expect that having excluded these patients would have increased the magnitude of difference in all comparisons done in aim one. This is a more plausible scenario in our opinion because patients tend to withhold sensitive information. However, since the baseline measures for these patients were similar to the patients included, we do not believe this tendency would create a differential effect. *Fourth*, the results present a population of patients with chronic pain from a single tertiary referral pain clinic; thus, further limiting generalizability of results to all patients with chronic pain.

## Conclusion

Our current study sought to better characterize patients with chronic who report using opioids and/or cannabis. We found that use of opioid and/or cannabis is associated with worse physical, psychological and social outcomes and higher healthcare utilization. However, it is important to characterize the trajectory of these patients in a prospective longitudinal study to better understand the causal relationships and identify important mediators and moderators. We can use this information to (1) target interventions aimed at improving the quality of life in those who suffer from chronic pain and (2) informing society of the true effects of opioids and/or cannabis on chronic pain.

## Data Availability

The data analyzed in this study is subject to the following licenses/restrictions: Deidentified data set can be provided upon request. Requests to access these datasets should be directed to Vafi Salmasi; vsalmasi@stanford.edu.

## Appendix

**Table A1:** Baseline demographics based on completion of cannabis questionnaire.

	Completion of Cannabis Questionnaire		*P*-Value
	No (N = 6313, 41.6%)	Yes (N = 8869, 58.4%)	
Age (Years);	52 ± 15.8	51.5 ± 16.8	<0.001
Mean ± Standard Deviation			
Sex; Number (%)			0.004
Female	4,058 (42.0%)	5,600 (58.0%)	
Male	2,164 (44.5%)	2,695 (55.5%)	
Missing	91 (22.3%)	317 (77.8%)	
Ethnicity; Number (%)			0.01
White	2,625 (34.8%)	4,926 (65.2%)	
Asian	331 (30.5%)	756 (69.5%)	
African-American	147 (34.0%)	285 (66.0%)	
Other	941 (36.2%)	1,657 (63.8%)	
Missing	2,269 (69.7%)	988 (30.3%)	
Emergency Room Visit; Number (%)			0.06
No	1,868 (28.8%)	4,610 (71.2%)	
Yes	1,212 (30.6%)	2,753 (69.4%)	
Missing	3,233 (72.1%)	1,249 (27.9%)	
Overnight Hospital Stay; Number (%)			0.05
No	2,457 (29.2%)	5,965 (70.8%)	
Yes	674 (31.3%)	1,478 (68.7%)	
Missing	3,182 (73.1%)	674 (31.3%)	
Number of Physician Visits; Mean ± Standard Deviation	6.5 ± 7.0	6.5 ± 7.9	0.6

**Table A2:** Pain intensity and PROMIS measures based on completion of cannabis questionnaire.

Mean ± Standard Deviation for all measure	Completion of Cannabis Questionnaire		*P*-Value
	No (N = 6313, 41.6%)	Yes (N = 8869, 58.4%)	
Ave. Pain Intensity (0–10)	5.7 ± 2.2	5.3 ± 2.3	<0.001
Worst Pain Intensity (0–10)	7.7 ± 2.1	7.3 ± 2.4	<0.001
Pain Catastrophizing Scale (0–52)	23.2 ± 12.7	21.5 ± 12.8	<0.001
PROMIS Pain Interference	65.0 ± 7.3	63.0 ± 8.0	<0.001
PROMIS Pain Behavior	59.2 ± 4.7	57.9 ± 5.7	<0.001
PROMIS Fatigue	60.3 ± 9.9	57.2 ± 10.5	<0.001
PROMIS Depression	55.5 ± 9.9	53.0 ± 10.0	<0.001
PROMIS Anxiety	56.8 ± 9.9	54.0 ± 9.9	<0.001
PROMIS Anger	51.1 ± 10.5	48.6 ± 10.3	<0.001
PROMIS Sleep Impairment	57.1 ± 9.9	55.2 ± 10.2	<0.001
PROMIS Sleep Disturbance	57.2 ± 9.5	55.5 ± 9.4	<0.001
PROMIS Social Isolation	47.9 ± 9.5	46.8 ± 9.5	<0.001
PROMIS Satisfaction with Social Roles and Activities	42.0 ± 9.5	43.5 ± 10.1	<0.001
PROMIS Emotional Support	50.8 ± 9.3	51.3 ± 9.5	0.003
